# Cx43 in Neural Progenitors Promotes Glioma Invasion in a 3D Culture System

**DOI:** 10.3390/ijms21155216

**Published:** 2020-07-23

**Authors:** Kanika Khosla, Christian C. Naus, Wun Chey Sin

**Affiliations:** Department of Cellular and Physiological Sciences, Life Sciences Institute, The University of British Columbia, Vancouver, BC V6T 1Z3, Canada; kanika2856@hotmail.com (K.K.); ccnaus@gmail.com (C.C.N.)

**Keywords:** Cx43, glioma, invasion, 3D, time-lapse imaging, human brain tumor-initiating cells

## Abstract

The environment that envelops the cancer cells intimately affects the malignancy of human cancers. In the case of glioma, an aggressive adult brain cancer, its high rate of recurrence after total resection is responsible for a poor prognosis. Connexin43 (Cx43) is a gap junction protein with a prominent presence in glioma-associated normal brain cells, specifically in the reactive astrocytes. We previously demonstrated that elimination of Cx43 in these astrocytes reduces glioma invasion in a syngeneic mouse model. To further our investigation in human glioma cells, we developed a scaffold-free 3D platform that takes into account both the tumor and its interaction with the surrounding tissue. Using cell-tracking dyes and 3D laser scanning confocal microscopy, we now report that the elimination of Cx43 protein in neural progenitor spheroids reduced the invasiveness of human brain tumor-initiating cells, confirming our earlier observation in an intact mouse brain. By investigating the glioma invasion in a defined multicellular system with a tumor boundary that mimics the intact brain environment, our findings strengthen Cx43 as a candidate target for glioma control.

## 1. Introduction

Cancer research has mostly focused on understanding and reversing the deleterious effect due to aberrant expression of mutated proteins in tumor cells. In this aspect, the gene of the ubiquitously expressed Cx43 (*Gja1*) is not usually altered in human cancers, based on a finding from a large cancer genome atlas (TCGA: https://www.cancer.gov/tcga). However, a TCGA query revealed preferential upregulation of Cx43 in malignant glioma [1], the most common brain cancer in adults, with very poor prognosis due to the high chance of recurrence, even after a successful total resection [2]. A report by Cottin and colleagues further determined that more than 10% of human gliomas displayed strong Cx43 immunostaining [3]. Although earlier work showed that the downregulation or absence of Cx43-mediated intercellular communication is associated with increased malignancy in tumor cells [4,5], it is now clear that Cx43 overexpression in tumor cells promotes invasion [4,6,7], especially in the presence of normal stromal cells such as astrocytes [8,9,10,11]. Indeed, there is strong evidence to suggest that Cx43 has a pivotal role in cancer of the central nervous system [12] and a promising therapeutic target for gliomas [13].

The microenvironment of cancer cells has gained prominence in driving tumor invasion [14]. Since one of the major functions of a gap junction is to mediate intercellular communication, Cx43 is well positioned to participate in microenvironment signaling. Cx43 protein levels are significantly enhanced in glioma-associated astrocytes, especially at the region adjacent to the tumor core and is crucial to the spreading of glioma cells at the invasive niche [15]. These findings suggest that Cx43 may modulate invasion via glioma–astrocyte communication. Indeed, in vivo studies with rats have found that glioma cells can increase their invasion by forming gap junctional intercellular communication (GJIC) with astrocytes [10,11]. Co-culture of U87MG human glioma cells with human astrocytes also enhances the invasive behavior of the glioma cells in a GJIC-dependent manner [16].

We previously demonstrated that elimination of Cx43 in astrocytes reduced glioma invasion in a syngeneic intracranial mouse model [15]. To extend this investigation on human patient glioma stem cells, we developed a scaffold-free 3D tissue culture model system [17] to examine the invasion of glioma cells into mouse progenitor cells cultured as spheroids and followed the invasion of human glioma cells in real time using cell-tracking dyes and confocal microscopy. We showed that human glioma cells exhibited reduced invasion into mouse spheroids when Cx43 protein was removed from the mouse progenitors. Our results from the 3D culture model confirmed our earlier observation in an intact mouse brain, strengthening the role of Cx43 in enhancing glioma invasion by being a conduit for the tumor cells to interact with the stromal environment.

## 2. Results

### 2.1. The Invasiveness of Human BTICs in 3D Culture Mirrored its Pathogenicity

**Figure 1:**
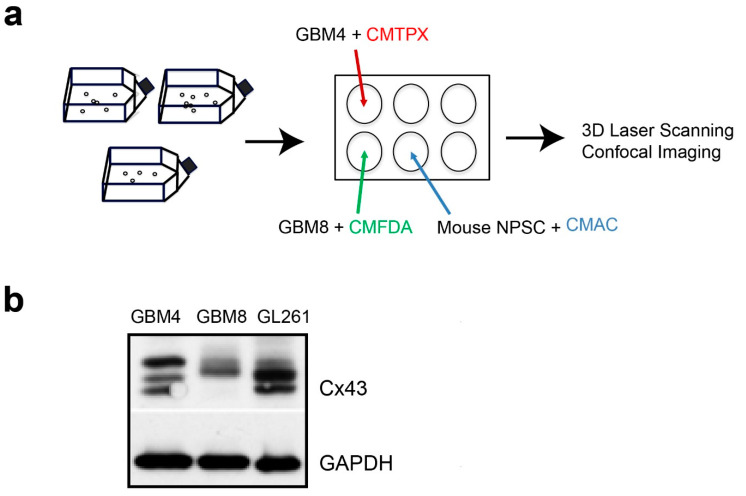
Cx43 protein is expressed in human brain tumor-initiating cells GBM4 and GBM8. (**a**) Schematic diagram illustrating the culture and labeling of spheroids with cell-tracker dyes (CMTPX, CMFDA and CMAC) to distinguish them for live imaging by confocal microscopy. (**b**) Western analysis of cell lysates of GBM4 and GBM8 with anti-Cx43 antibody showing multiple protein bands corresponding to different phosphorylated isoforms. Cx43 species in mouse GL261 glioma cells used in our intracranial mouse implantation [15] was included as a comparison. GAPDH was used as loading control.

Cancer stem cells are key drivers of tumor progression and invasion [18,19]. Therefore, we first characterized 2 lines of human brain tumor-initiating cells (BTICs), GBM4 and GBM8, which have been propagated as spheroids in serum-free medium [20]. GBM4 is less invasive of the two lines, with nodular pathology and massive endothelial proliferation [20]. In contrast, GBM8 is very invasive, with a PNET-like pathology [20]. Multiple Cx43 protein bands that corresponded to different phosphorylated isoforms [21,22] were detected in the cell lysates of both BTICs (Figure 1b). The smallest Cx43 band detected in GBM4 was absent in GBM8 but present in GL261, a mouse glioma cell line that was used in our intracranial mouse glioma model [15]. Taken together, the results indicated that these BTICs have the potential to form Cx43 channels with each other and with adjacent normal cells.

**Figure 2:**
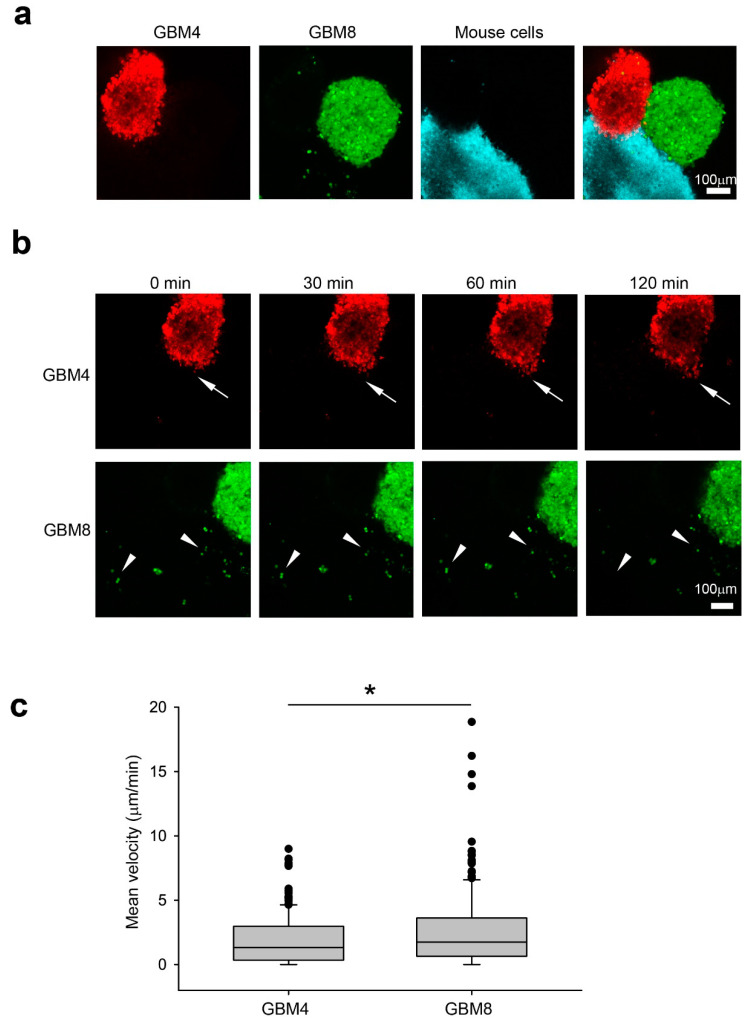
Invasiveness of GBM4 and GBM8 correlate with their pathogenicity in human patients. (**a**) Co-culture of GBM4 (red), GBM8 (green) and C57BL6 mouse neural progenitor cell derived spheroids (blue) in Noble agar before imaging. Scale bar 100 μm (**b**) Representative time-lapse images showing GBM8 preferentially invaded as single cells (white arrowheads) while GBM4 invaded in a collective manner as a group (white arrows). Images were taken 10 min apart at a 5 µm step size using confocal microscopy. Scale bar 100 μm (**c**) Mean velocity of GBM4 and GBM8 single cells in mouse wild-type spheroids. The data shown here are pooled from at least three experiments. Data were analyzed by Student’s t test. * *P* < 0.05.

We examined their invasiveness in a 3D human glioma model [17] by measuring the migration distance of GBM4 (red) and GBM8 (green) in wild-type (WT) mouse progenitor (blue) spheroids Figure 1a, Figure 2a). After 2 h of co-culture, isolated single GBM8 cells (white arrowhead) were detected in the mouse spheroids (Figure 2b). In contrast, GBM4 preferentially invaded mouse spheroids in a collective manner (white arrow) while maintaining contact with neighboring cells (Figure 2b). Indeed, GBM8 cells exhibited higher velocity (2.657 μm/min +/- 0.212, *n* = 195) than GBM4 cells (1.928 μm/min +/- 0.150, *n* = 171) in WT spheroids (Figure 2c). Taken together, our results from the 3D spheroid invasion platform are in agreement with their invasiveness in a human patient.

### 2.2. The Absence of Cx43 in Mouse Progenitor Cells Reduces Glioma Invasion

**Figure 3:**
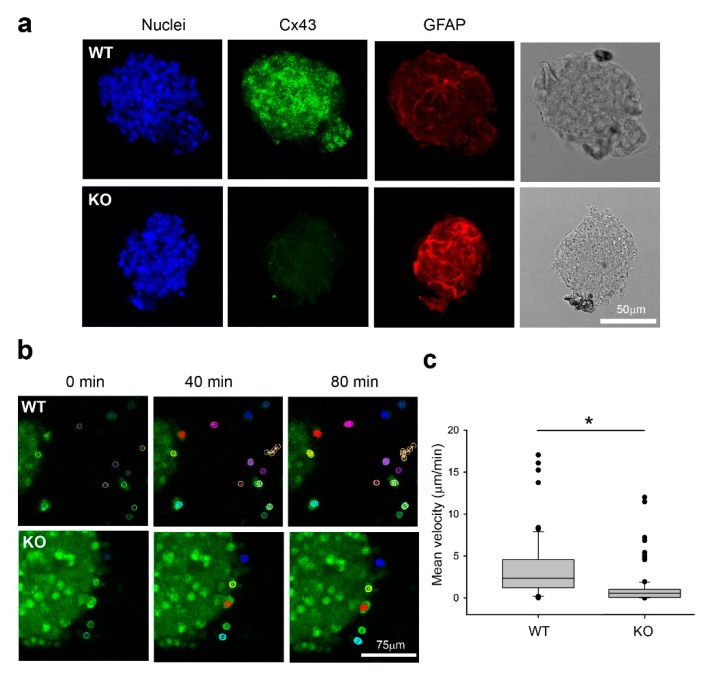
Elimination of Cx43 in mouse progenitor cells reduces GBM8 invasion. (**a**) Wild-type (WT) and Cx43 knockout (KO) spheroids were co-stained with anti-Cx43 and anti-GFAP antibodies. GFAP was detected in both WT and KO spheroids. In contrast, the punctate staining of Cx43 was only detected in WT but not the KO spheroids, confirming successful knockout of Cx43 in the mouse progenitors. Scale bar 50 μm (**b**) 3D cell tracking was carried out using the Fiji plugin, MTrackJ. Representative images showing cell movement in two 40-min periods. The track of a single cell is denoted by a colored lane in a 3D projection. Each circle represents the x,y,z location of the cell at a time point from 0 to 40 min, and from 0 to 80 min. The difference between 40 and 80 min track illustrates the movement of cells during this time interval. Note that the movement of a cell in the z direction will not be apparent in these 2D representative images. Scale bar 75 μm (**c**) Mean velocity of GBM8 in WT and KO mouse spheroids. The data shown here are pooled from at least three experiments. Data were analyzed by Student’s t test. * *P* < 0.05.

In order to validate our 3D invasion model platform, we made use of mouse WT and Cx43 knockout (KO) cells from our earlier study showing that elimination of Cx43 in astrocytes reduces glioma invasion in an intact mouse model [15]. We first examined the characteristics of mouse WT and Cx43 KO spheroids with antibodies against glial fibrillary acidic protein (GFAP) and Cx43, both of which are highly expressed in reactive astrocytes induced by a brain lesion [23]. GFAP was detected in both WT and KO spheroids (Figure 3a), which is in agreement with our previous observation that abolition of Cx43 in astrocytes in vivo does not affect GFAP expression [15,23]. In contrast, the punctate staining of Cx43 was only detected in WT but not the KO spheroids (Figure 3a), confirming successful knockout of Cx43 in the mouse progenitor cells. We decided to examine only GBM8 co-cultured with WT and KO spheroids in this series of experiments due to its increased invasiveness compared to GBM4 (Figure 2). After co-culture for 2 h, there were more GBM8 cells in the WT mouse spheroids than in KO spheroids (Figure 3b). When we measured the velocity of GBM8 cells that had invaded into the WT spheroid after exiting from the BTICs spheroid, we found that GBM8 moved significantly slower in KO (1.083 μm/min +/- 0.205, *n* = 102) than in WT (3.495 μm/min +/- 0.483, *n* = 102) spheroids (Figure 3c). Therefore, as demonstrated previously in the intracranial mouse glioma model [15], the absence of Cx43 in normal cells affects the invasiveness of tumor cells, strengthening a critical role of Cx43 in the microenvironment.

## 3. Discussion

The tissue environment that envelops the glioma cells not only affects the dissemination of cancer cells, but it also influences the susceptibility of cancer cells to therapeutic intervention [24]. One prominent feature of the glioma microenvironment is the presence of a large number of reactive astrocytes exhibiting increased GFAP and Cx43 expression in the peri-tumor region [15,25,26,27]. In this environment, Cx43-mediated GJIC is expected to occur between glioma cells, between astrocytes and heterocellularly between glioma cells and astrocytes. In the latter case, Cx43 has been shown to be a facilitator in tumor invasion by allowing transfer or exchange of signaling molecules such as miRNA or cGAMP between cancer cells and astrocytes [16,28].

An early study indicates that 90% of recurrent gliomas occur within 2 cm of the resected tumor [29]. Therefore, we developed a 3D platform with the capacity to quickly quantify the invasion of patient-derived glioma cells into normal tissue, focusing on the invasive niche [30] at the tumor–stromal interface. In addition, a tumor organoid culture will have the advantage of having cellular complexities that better assess therapeutic response [31]. As a proof-of-principle study, we demonstrated that the absence of Cx43 in mouse progenitor cells is sufficient to affect human BTICs invasion in a 3D culture system.

Cx43-dependent signaling pathways in the tumor microenvironment are emerging to be attractive anti-cancer targets. Carbenoxolone, a widely used Cx43 gap junction inhibitor, enhanced glioma cell death and survival of treated mice by 27% [32]. Meclofenamate is a promising orally bioavailable therapeutic that inhibits gap junction-mediated processes [28] and is now being tested in human patients with recurrent or progressive brain metastasis (https://clinicaltrials.gov/ct2/show/NCT02429570). Recently, we reported that the application of a TAT-Cx43_266-283_ peptide that mimics the effect of Cx43 on c-Src inhibition reduces the growth and invasion of human glioma stem cells in immunodeficient mice, without adversely affecting neurons and astrocytes [33]; similarly, the TAT-Cx43 peptide reduces the invasiveness of mouse glioma cells GL261 in an immunocompetent mouse model [33].

Increasing evidence suggests that somatic alterations of the genome in host tissue contribute to tumor invasion [34,35]. A co-culture of patient-derived glioma cells with normal cells from the same patient in a 3D system will better mimic glioma invasion in an intact brain. Therefore, having determined that the absence of Cx43 reduces glioma invasion in our 3D model, our next objective is to examine the efficacy of Cx43 inhibitors with normal spheroids generated from human-induced pluripotent stem cells. Instead of using confocal microscopy to track invasion, a high content array image scanner will greatly increase throughput by screening multiple drug combinations on glioma samples. Our platform will potentially have a wide application to assess the efficacy of cancer treatment with matching patient tumor and normal spheroids of different types of human cancers, thereby facilitating the translation to personalized oncology.

## 4. Materials and Methods

### 4.1. Generation of Cx43 Knockout Mouse Progenitor Cells

Cx43 conditional knockout mice were generated by crossing mice containing GFAP-Cre [36] with C57BL mice harboring floxed Cx43 alleles [37]. Mice of either sex were used in the experiment, maintained in an animal facility for a 12 h light/dark cycle and were provided food and water ad libitum. All breeding and animal procedures were approved by The University of British Columbia Animal Care Committee (Protocol No: A09-0847) and performed in accordance with the guidelines established by the Canadian Council on Animal Care. Mouse neural progenitor stem cells were isolated from mice, similar to the procedure described in [38] and modified as described in [39]. Briefly, cortices freed of meninges were dissociated with 0.25% trypsin-EDTA at 37 °C for 5 min. The digested cortices were washed twice with DMEM supplemented with 10% FBS and gently triturated in DMEM without FBS with a p200 pipette tip. After pelleting, the cell suspension was resuspended in DMEM/F12 (Life Technologies, Carlsbad, CA, USA) and passed through a 40 μm cell strainer (BD Biosciences, San Jose, CA, USA) and plated at 2 × 10^4^/cm^2^ in complete NeuroCult media (STEMCELL Technologies, Vancouver, BC, Canada) supplemented with 20 ng/mL rhEGF (STEMCELL Technologies, Vancouver, BC, Canada), 10 ng FGF (STEMCELL Technologies, Vancouver, BC, Canada) and 0.0002% Heparin (STEMCELL Technologies, Vancouver, BC, Canada). Human BTICs, GBM4 and GBM8 [20,40] were cultured in the same media. Spheroids were subcultured with Accutase (STEMCELL Technologies, Vancouver, BC, Canada) according to the manufacturer’s instructions.

### 4.2. Preparation of Spheroids for Imaging

To generate spheroids for the invasion experiments, 10^5^ isolated cells were plated in a 6-well plate with gentle rotational shaking at approximately 34 rpm and allowed to form spheroids for 2 d in the presence of either CellTracker CMTPX (Red dye, Invitrogen, Carlsbad, CA, USA), CMAC (Blue dye, Invitrogen, Carlsbad, CA, USA) or CMFDA (Green dye, Invitrogen, Carlsbad, CA, USA), respectively (Figure 1a). Combination was achieved by transferring labeled spheroids into a 2.0 mL Eppendorf tube using a sterile plastic eyedropper, washing once in PBS, then combining the spheroids of interest at the bottom of a 1.5 mL Eppendorf tube. The spheroids were allowed to settle at the bottom of an Eppendorf tube followed by a 1 h incubation at 37 °C to allow contact adhesion to occur. For live cell imaging, aggregates were mounted in 0.8% Noble agar/PBS that had been cooled to 39 °C prior to imaging in a 35 mm glass- bottom dish (MatTek Corporation, Ashland, MA, USA). A 37 °C heater was used to warm the imaging chamber, beginning at least 3 h prior to the start of live cell imaging. Aggregated spheroids were imaged at single cell resolution with a Leica TCS SP5 II Basic VIS confocal system (Leica Microsystems, Wetzlar, Germany) at a time interval of 10 min for 2 h.

### 4.3. Analysis of Spheroids Invasion

Images were analyzed, including color merging, maximal projections and 3D reconstructions using ImageJ (version 1.48v) software [41]. Individual BTICs migrating away from the spheroid into the C57BL mouse neurospheres were tracked using the MTrackJ plugin [42].

### 4.4. Immunofluorescence of Self-Assembled Spheroids

Mouse spheroids of less than 100 μm in diameter were fixed with 4% paraformaldehyde in PBS for 30 min at room temperature with gentle inversion, followed by three washes of PBS. The spheroids were blocked for 1 h at room temperature in 2% BSA and 0.3% Triton X-100, incubated overnight at 4 °C in 1% BSA and 0.1% Triton X-100 with rabbit anti-Cx43 (1:200, Sigma-Aldrich, St. Louis, MO, USA) and mouse anti-GFAP (1:100, Sigma-Aldrich, St. Louis, MO, USA), probed with Alexa Fluor-conjugated secondary antibodies (1:500, Invitrogen, Carlsbad, CA, USA), mounted in Prolong Antifade mounting media with 4′-6-diamidino-2-phenylindole (DAPI) (Invitrogen, Carlsbad, CA, USA) and visualized with a Leica TCS SP5 II Basic VIS confocal system.

### 4.5. Protein Isolation and Western Analysis

GBM4 and GBM8 cell pellets were lysed in a buffer containing 0.1% SDS, 1% IGEPAL, 0.5% Sarkosyl, 50 mM Tris-HCl (pH 8.0), 150 mM NaCl supplemented with protease inhibitors (Roche Applied Science, Penzberg, Germany) and phosphatase inhibitors (Sigma-Aldrich, St. Louis, MO, USA). Rabbit anti-Cx43 (1:3000, Sigma-Aldrich, St. Louis, MO, USA) and anti-GAPDH (1:10,000, Hytest, Turku, Finland) were used as primary antibodies, followed by either anti-rabbit-HRP or anti-mouse-HRP (Sigma-Aldrich, St. Louis, MO, USA) as secondary antibodies. Protein bands were detected with Amersham ECL western detection reagents (GE Healthcare, Chicago, IL, USA).

### 4.6. Statistical Analysis

SigmaPlot version 13.0 software (San Jose, CA, USA) was used for statistical analysis. The results are presented as mean +/- SEM. Data were analyzed by Student’s t test. *P* < 0.05 was considered significant.
